# Radioneuromodulation by Dual-Target Irradiation in Pain Crisis From Trigeminal Neuralgia

**DOI:** 10.7759/cureus.20971

**Published:** 2022-01-05

**Authors:** Eduardo E Lovo, Alejandra Moreira, Kaory C Barahona, Victor Caceros, Claudia Cruz, Juan Arias

**Affiliations:** 1 Neuro-radiosurgery, International Cancer Center, Diagnostic Hospital, San Salvador, SLV; 2 Neurosurgery, International Cancer Center, Diagnostic Hospital, San Salvador, SLV; 3 Radiation Oncology, International Cancer Center, Diagnostic Hospital, San Salvador, SLV; 4 Pain Management, International Cancer Center, Diagnostic Hospital, San Salvador, SLV

**Keywords:** functional neurosurgery, neuromodulation, pain, radiosurgery, trigeminal neuralgia

## Abstract

Background

Radioneuromodulation (RNM) can explain the immediate pain relief experienced by a subgroup of patients after stereotactic radiosurgery (SRS) for trigeminal neuralgia (TN). In this study, our main objective was to demonstrate that a minimum of a 50% reduction in TN pain can be achieved consistently in under 72 hours by targeting the affected nerve, the contralateral centromedian nucleus, and parafascicular complex in patients experimenting a prolonged refractory pain crisis.

Methodology

We treated eight patients experiencing severe TN pain crisis in whom percutaneous procedures had failed or were unwanted with SRS with an intention to procure pain relief in under 72 hours. The affected trigeminal nerve was targeted using a 4-mm collimator with an 80 to 90-Gy dose; an additional target was defined in the mesial portions of the thalamus and irradiated using the 4-mm collimator with a 120 to 140-Gy dose.

Results

The median duration of TN was 60 months, the median duration of pain crisis was 10.7 weeks despite the best medical treatment, and the mean presenting visual analogue score (VAS) was 10 at the time of treatment. The median follow-up was 135 days (range, 65-210). Twenty-four hours after treatment, two (25%) patients had no pain (VAS 0), three (37.5%) had mild pain (VAS 1 to 3), and three (37.5%) had moderate pain (VAS 4 to 7). Forty-eight hours after treatment, all patients reported pain relief, seven (87.5%) reported >50%, and one (12.5%) patients reported 30% relief. The three-month median VAS score was 3 (range, 0 to 5). At the last follow-up, there were no adverse events to report.

Conclusions

Dual irradiation to the affected trigeminal nerve and contralateral mesial structures of the thalamus may provide fast pain relief for patients experiencing a prolonged pain crisis from TN, which veers away from the concept that the SRS pain relief effect is generally delayed and holds no place in the management of such patients. Although this is a small series with a limited follow-up duration, no adverse effects were noted.

RNM can be defined as the capacity to alter neuronal activity through targeted delivery of a stimulus of radiation at a duration too brief to be explained by the development of a focal lesion. The immediate pain relief and its habitual oscillatory nature of lesser pain recurrence in most patients until enough time elapses for pain stabilization clinically demonstrates that the pain circuitry is altered and remains functional, thus accomplishing a neuromodulation effect even at the face of an apparent doses suspected to be ablative. Further research is needed to understand if this clinical effect is achieved with a suspected sub-ablative dose.

## Introduction

Neuromodulation is the alteration of neuronal activity through the targeted delivery of an electrical or chemical stimulus. It can be achieved through minimally invasive techniques (e.g., deep brain stimulation) or noninvasively by repetitive transcranial stimulation to a larger area of the brain [1,2]. An alleged benefit of neuromodulation is that it is potentially reversible. This procedure does not require generating a permanent lesion in the neuronal circuitry similar to other procedures such as radiofrequency ablation, high-frequency ultrasound, or what is classically attributed as the mechanism of action of stereotactic radiosurgery (SRS) [3-7].

Régis et al. proposed that SRS could produce a nonablative neuromodulation effect in epilepsy patients and other pathologies where radiological evidence failed to demonstrate a lesion, but the therapeutic effect remained [8,9]. Empirically, functional SRS practitioners have consistently witnessed a clinically positive effect either in pain management for oncological pain, or intermittently in trigeminal neuralgia (TN), epilepsy, or tremor symptoms immediately following treatment or shortly thereafter [10-15]; a duration too brief for our current understanding of the therapeutic effect of late tissue response to radiation [16].

Ideally, radioneuromodulation (RNM) achieves a long-lasting therapeutic effect or alteration of viable neurons’ function at a dose that would not produce tissue ablation via focalized radiation [17]. Therefore, the threshold of radiation doses should be lower than the typical doses suspected to generate lesions (i.e., above 130 Gy) [16]; however, possibly more important than if a subchronic or chronic late tissue permanent alteration is created or not is the achievement of an immediate or fast-acting clinical benefit in the majority of patients regardless of the dose.

Refractory TN, especially in patients experiencing a pain crisis, can become a multidisciplinary challenge. Most patients with a long history of pain progression are usually heavily medicated or are medicated by multiple first-line or second-line regimens. Many of these patients also receive invasive treatment modalities such as percutaneous rhizotomy or microvascular decompression [18].

Gamma Knife is a safe and effective minimally invasive treatment modality commonly used to treat medically refractory TN [19]. Its high-precision ability to target small areas in the brain contributes to pain alleviation in more than 80% of patients. Targeting the trigeminal nerve to produce pain relief has been widely studied. Latency to pain relief varies from 15 to 80 days, with an average of 30 days where most patients report pain relief. The maximum time to achieve optimum pain relief can range from three to six months [18,19]. Our clinical experience and that of others suggest that 16-20% of patients can experience permanent or temporary pain cessation or relief within 24 hours after SRS [10,20], and it remains unclear why this effect is only noted in some patients. In a previous study, we treated a different set of patients with refractory TN via a single 4-mm session of high-dose radiation to the contralateral centromedian (CM) nucleus and parafascicular complex (PFc) of the thalamus [11]. We saw transitory pain relief in less than eight days in most patients (60%), although pain recurred in almost all of them with varying pain levels until the three-month evaluation when pain relief was finally achieved [11]. Our current understanding of radiobiology attributes a treatment effect after irradiating the thalamus (thalamotomy) after 90 days, which is when the late effect of high-dose radiation is expected to generate a lesion [16].

Schneider et al. postulated a concept for RNM that alters neural activity if radiation is focally delivered to brain tissue at subablative doses [17]. Therefore, RNM can also explain the prompt pain relief (indicated by a reduction in the visual analog scale [VAS] score) that usually occurs within 72 hours after treatment, even with suspected ablative doses as the duration is too short to explain the beneficial clinical effect. Based on this principle, we hypothesized that TN patients in a pain crisis could experience fast pain relief if the affected trigeminal nerve and the contralateral CM and PFc were treated simultaneously using SRS based on our RNM experience when these structures were irradiated separately [10,11].

This study investigated the RNM pain-relieving effect within 72 hours using SRS in refractory TN patients in a pain crisis. To our knowledge, this is the first series to deliberately elicit the RNM effect to alleviate pain using multiple targets and in areas other than the hypophysis.

## Materials and methods

Patient selection

We analyzed a small group of patients with refractory TN who were experiencing a severe pain crisis at the time of treatment. The study included patients aged 18 years or older who reported a VAS of 10 at the time of treatment. All patients had been declared refractory to medical treatment, including opioids, were unaffected by other invasive treatment modalities, and no other therapy was feasible or desired. The primary endpoint was to achieve an RNM effect within 72 hours of treatment by reducing the VAS score by at least 50%. VAS 0 indicated no pain, VAS 1 to 3 was considered mild pain, 4 to 7 was moderate pain, and 8 to 10 was intense pain. Pain measurement was based on the quality of pain assessment reporting [21], and paresthesia, as a complication, was based on Barrow Neurological Institute (BNI) facial numbness score.

Additionally, patients’ pain crises had to be constant for at least four weeks and without adequate pain control via regimens prescribed by a specialist (e.g., pain management medicine specialist, neurologist, or neurosurgeons). Moreover, included patients needed to have sufficient family support to understand treatment expectations, evaluate VAS changes after SRS, and report changes during the follow-up period. On the day of the treatment, patients were asked about their TN pain history including time of pain since diagnosis, exact pain location, VAS at the time of SRS, BNI prior to treatment and at last follow-up, and history of previous treatments including procedures and medication use. Patients were asked to continue using their regular medicine as prescribed before SRS, and their regimens were later adjusted depending on their response to SRS. The study was approved by the institution’s ethical committee, and all patients provided written consent.

Radiosurgical technique

**Figure 1:**
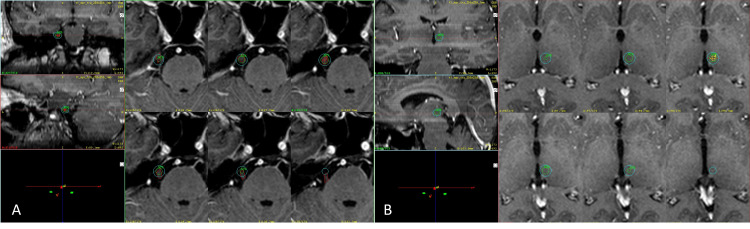
Final plan with dual targets. A T1 three-dimensional view of a final plan. (A) Coronal, sagittal, and axial views of the 4-mm shot placed in the right trigeminal nerve in the retrogasserian area. (B) Coronal, sagittal, and axial views of the 4-mm shot placed in the contralateral mesial regions of the thalamus. The green isodose line corresponds to 50% of the dose prescribed; the light blue isodose line corresponds to the dose gradient or 25% of the dose prescribed.

On the day of the procedure, patients were placed under local anesthesia and slight sedation, and a stereotactic frame (Masep Medical Company, Shenzhen, China) was placed by a neurosurgeon with the aid of ear bars searching for frame parallelism to the anterior and posterior commissure line or the intercommissural line (ICL). Magnetic resonance imaging (MRI) was performed using a 1.5-Tesla Avanto (Siemens Corporation, Erlangen, Germany) as a T1 and T2 constructive interference in steady-state of 1-mm slices with no space covering the thalamus to the superior border of the corpus callosum and below the trigeminal nerve. Images were later transferred to the treatment planning station. For treatment planning, the anterior commissure and posterior commissure (PC) were identified in the axial plane, the images were then transformed into a sagittal plane using fusion tools, and the ICL was drawn between the T1 and T2 sequences. The distance from the PC was taken anteriorly along the ICL (usually 4 mm); this measurement was tagged as Y. A 90° angle was traced from the PC to the Y along the ICL, and Z was usually determined 4-5 mm above the ICL. Images were then reoriented to axial views, and X coordinates were 5-6 mm lateral from the contralateral thalamic border to the side of the pain. Using a 4-mm collimator, a single shot was placed, and a 120 to 140-Gy dose was delivered. Finally, using T1-weighted multiplanar gradient recall gadolinium with 1-mm slices in axial orientation, a single 4-mm isocenter positioned at the retrogasserian zone (RGZ) of the affected trigeminal nerve with an 80 to 90-Gy dose was prescribed. Plans were reviewed and approved by the radiation oncology and neurosurgery teams (Figure 1).

Patient follow-up

Patients were contacted 24, 48, 72, and 96 hours after treatment and every 15 days for the next three months. RNM effect was defined as a prompt relief of pain (i.e., VAS score reduction of ≥50%) specifically within 72 hours of SRS. Patients were asked to assess their pain during every follow-up call and report any increase in pain despite the use of their usual medication.

## Results

From January 2021 to September 2021, eight (six [75%] women, two [25%] men) patients were eligible for the treatment protocol and follow-up. The mean patient age at treatment was 63.2 years (range, 41 to 85 years; Table 1).

**Table 1:** Patient and treatment characteristics. TN: trigeminal neuralgia; CNV1: cranial verve V–ophthalmic branch; CNV2: cranial nerve V–maxillary branch; CNV3: cranial nerve V–mandibular branch; VAS: visual analogue pain scale; BNI: Barrow Neurological Institute Pain scale; RF: radiofrequency; PMC: percutaneous microballoon compression; MVD: microvascular decompression; NSAIDs: nonsteroidal anti-inflammatory drugs Refused: patients refused other types of invasive treatments.

Patient	Age	Sex	Duration of pain (months)	Duration of crisis (weeks)	Primary or secondary TN	Type of medications used	Type of previous treatments	Affected side	Affected branch	Thalamotomy	Dose to nerve and thalamus	VAS prior to treatment	BNI prior to treatment
1	60	F	13	8.5	Primary	NSAIDs, neuromodulators	RF PMC	Right	CNV_1_, CNV_2_, CNV_3_	Left	90/140 Gy	10	5
2	57	F	72	10.5	Primary	Neuromodulators	MVD	Left	CN_2_, CNV_3_	Right	90/140 Gy	10	5
3	78	F	48	12	Primary	NSAIDs, neuromodulators	Refused	Right	CNV_1_, CNV_2_, CNV_3_	Left	90/140 Gy	10	5
4	85	M	19	5.5	Primary	Neuromodulators, opioids	Refused	Right	CNV_2_, CNV_3_	Left	90/140 Gy	10	5
5	41	M	144	8.5	Primary	NSAIDs, neuromodulators	Refused	Right	CNV_1_, CNV_2_, CNV_3_	Left	90/140 Gy	10	5
6	60	F	36	11	Primary	Neuromodulators	RF PMC	Right	CNV_1_, CNV_2_, CNV_3_	Left	90/140 Gy	10	5
7	69	F	120	16	Secondary	Neuromodulators	RF	Right	CNV_1_, CNV_2_,	Left	90/140 Gy	10	5
8	56	F	144	20	Primary	NSAIDs, neuromodulators	RF PMC	Right	CNV_1_, CNV_2,_ CNV_3_	Left	80/120 Gy	10	5

The median duration of pain was 60 months (range, 13 to 144 months), and the median duration of the pain crisis was 10.7 weeks (range, 5.5 to 16 weeks). The most affected side was the right side in seven (87.5%) patients, and all patients presented pain in more than one of the trigeminal branches (CNV1, CNV2, CNV3). One (12.5%) patients had postherpetic TN in CNV1, and all other patients (87.5%) had primary TN.

Regarding previous treatments, one (12.5%) patients had received a microvascular decompression of the trigeminal nerve and experienced pain relapse within a few months after the procedure. Four (50%) patients had received percutaneous radiofrequency procedures, and the other three (37.5%) had received medical treatment with primary line drugs (neuromodulators), nonsteroidal anti-inflammatory drugs (NSAIDs), or opioids. Two of these three (66%) patients declined other forms of invasive procedures (e.g., balloon rhizotomy) due to age and comorbidities. Presenting VAS score at the time of treatment was 10; thus, all patients presented with a BNI of 5. The median follow-up was 135 days (range, 65 to 210 days).

**Figure 2:**
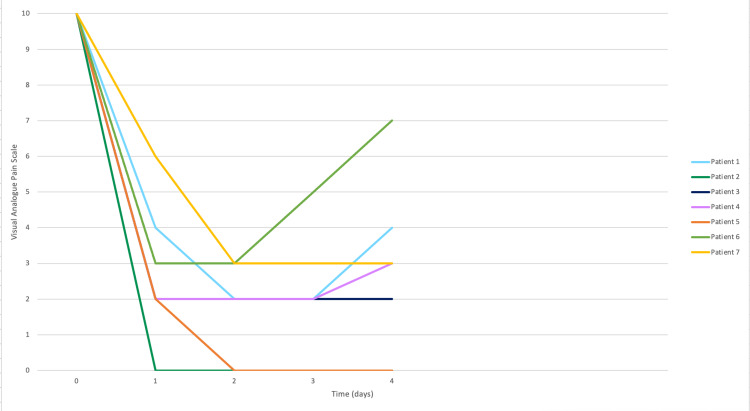
Pain progression and radioneuromodulatory effect after 96 hours of radiosurgery.

At 24 hours after treatment, two (25%) patients had no pain (VAS 0), three (37.5%) reported mild pain (VAS 1 to 3), and three (37.5%) had moderate pain (VAS 4 to 7). At 48 hours, all patients reported some level of pain relief (100%), seven (87.5%) had more than 50% relief, and one (12.5%) patient reported 30% pain relief according to VAS (Figure 2).

**Figure 3:**
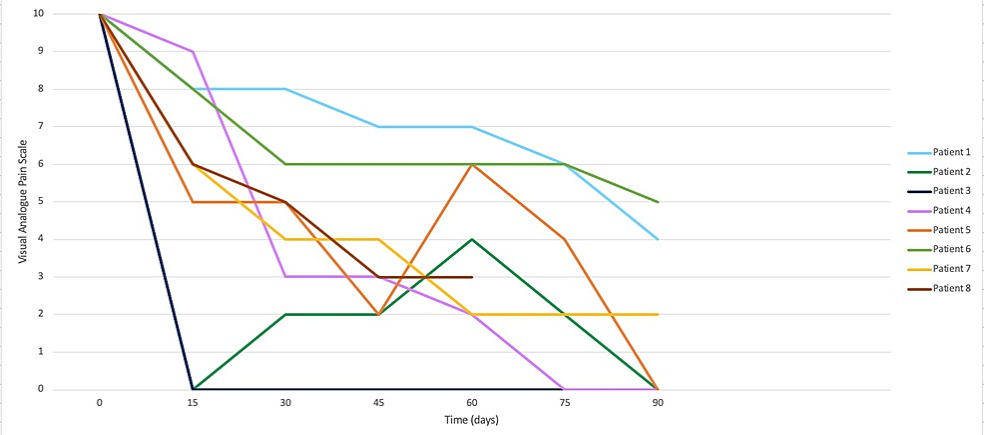
Pain progression at 90 days of treatment.

Overall, one (12.5%) patients experienced increased pain with a VAS 4 at 48 hours, VAS 5 at 72 hours, and VAS 7 at 96 hours. All other patients (87.5%) maintained pain levels between VAS 0 and VAS 5 within 96 hours of treatment. The RNM effect was seen in all patients 48 hours after treatment, with a mean VAS of 2 (range, 0 to 3). The pain relief effect was monitored for at least 60 days after treatment (Figure 3); one patient reported a VAS of 0 after five days of treatment with no change in pain as of the last follow-up. Another patient had an RMN effect 96 hours after treatment but had small relapses of pain that gradually decreased over time until three months postoperatively.

In total, three (37.5%) patients presented with short pain crises (VAS 8 to 10) within the follow-up period and were managed with other medications.

The median score at three months was VAS 3 (range, 0 to 5). Three months after treatment, one (12.5%) patient had an excellent response (BNI I), and seven (87.5%) patients had good results (BNI IIIa, IIIb). The total success rate per BNI score was 100% (BNI I to IIIb). Two patients (25%) had moderate pain scores at the last follow-up (VAS 4 to 7), but they confirmed pain alleviation with complementary medication use. Six (75%) patients reported no pain to mild pain in their last follow-up examination. No complications, including facial paresthesia, were documented.

## Discussion

Treatments for TN are generally divided into the following three categories: medical treatment, surgical treatment (e.g., microvascular decompression, percutaneous rhizotomy), and SRS. Craniotomy and microvascular decompression emerged as the gold standard treatment for medically refractory TN by the end of the 20th century [22]. Unfortunately, some patients with TN are poor surgical candidates due to medical comorbidities, or they do not want to undergo invasive treatments. Additionally, not all procedures, including microvascular decompression, yield complete pain relief among all candidates. The role of SRS in the management of refractory TN has expanded as it is the least invasive treatment modality. The duration to achieving pain relief when irradiating the TN varies from immediate to six months post-SRS, but a few patients experience persistent pain or a quick relapse [23-25]. Although some studies have reported immediate pain relief following SRS [20], the mechanism is poorly understood.

Peripheral transmission and central areas of pain integration participate in the complex mechanisms regarding severe attacks of TN, which may account for the difficulty of treating refractory TN with a single target approach. Thalamotomy using SRS dates to the origins of Gamma Knife SRS with Leksell [26]. This bloodless operation could potentially alleviate pain without loss of sensation. The preferred neurosurgical pain targets are the CM and the PFc region, especially in trigeminal and facial pain. The time to pain relief ranges 2-210 days, with 40-60% success after irradiating this area with doses that are typically above 130 Gy [11,27,28].

The rationale for targeting the TN, the contralateral CM nucleus, and PFc of the thalamus lies in the anatomy and pathophysiology of facial pain. The ventral posteromedial nucleus is adjacent to the CM-PFc region and is a relay center for the main sensory nucleus and the spinal nucleus of the trigeminal nerve that receives direct afferents from the spinal tract. The CM-PFc region also receives afferents from the ventral posterolateral and spinothalamic tract and the trigeminal lemniscus and finally gives efferent connections to the anterior cingulate cortex. This might explain the affective aspects regarding pain perception and, possibly, the RNM effect when this area is irradiated. Peripheral targets in the trigeminal nerve vary; some studies targeted the dorsal root entry zone (DREZ), where the anatomical transition in myelin characteristics is present, and others targeted the RGZ. In our experience, targeting the RGZ was more effective in pain control than the DREZ with similar adverse events, and the RNM effect was most often seen when targeting the RGZ. However, the maximum therapeutical effect was noted three months after treatment [11].

Due to its delayed therapeutical effect, SRS does not traditionally play an important role in TN acute pain management. The results of this small series challenge the classical perception and open the possibility of SRS as an acute pain management approach for non-oncological pain such as refractory TN. RNM or most likely radio-neuro-endocrine-modulation has been documented in 70-80% of terminally ill oncology patients with refractory pain from bone metastases. Pain relief (as >50% VAS reduction) often occurs in less than 72 hours when a high dose of radiation is delivered to the hypophysis [12-14]. Giving a high dose of radiation to the body’s main gland could mediate a fast response via hormones, which is why it is important to document a dependable, fast pain relief effect when irradiating different structures in the brain that do not produce hormones such as the medial structures in the thalamus.

Despite achieving rapid pain relief after SRS in all patients, some experienced recurrent pain of different intensities that required additional pain management with NSAIDs, and two patients required an occasional intravenous phenytoin bolus (500-600 mg). Although our series is too small to assess the impact of these additional medical treatments, it indicates that the RNM effect does not result in complete pain relief for all patients, and possibly clinically proving that the neuronal circuitry responsible for pain transmission even at expected ablative doses remains viable but its function is altered and modulated. Physicians should monitor patients closely during additional pain management treatments until they achieve a permanent response, which can take up to three months in our experience.

Regarding safety, no patient experienced facial numbness as of this writing, but additional follow-up is necessary. Irradiating the mesial structures of the thalamus is safe; Urgosik et al. and our group found no neurological disturbances associated with this procedure [11,27], along with absence of complications from this small series the possibility of this being a safe approach is reinforced. Nevertheless, this needs to be further studied in prospective clinical trials.

In animal studies for epileptic models, high doses of radiation delivered with a Gamm Knife have shown a decrease of up to 55% of neuronal firing compared to the control animals; the rats receiving <50 Gy had no signs of necrosis [29]. Smaller doses slow neuronal transmission, while higher doses impair neuronal activity. The RNM effect noted in our series may be due to both phenomena, with an initial almost immediate decrease in the neuronal transmission of pain (thus a favorable clinical outcome) and a later (possible) impairment of neuronal activity.

The doses used in this study can be reduced, especially to the nerve, as other groups reported similar outcomes using a lower dose to the nerve (80 Gy) [26]. This has been our approach because in patient eight doses were lowered to 120 Gy to the mesial structures and the thalamus and 80 Gy to the nerve with similar therapeutic effect. Nevertheless, a greater patient volume is needed to demonstrate similar effect using a lower dose to the medial structures.

Our study was limited by its small number of patients; however, it provides a clinical proof of concept that speaks to the potential for SRS to treat functional diseases with a multitarget approach in appropriate patients.

## Conclusions

Dual irradiation to the affected trigeminal nerve and contralateral mesial structures of the thalamus may provide fast pain relief for patients experiencing a prolonged pain crisis from TN, which veers away from the concept that the SRS pain relief effect is generally delayed and thus no place in the management of such patients. Although this is a small series with a limited follow-up duration, no adverse effects were noted. From our previous experience and this recent series, the combination of targets might be a safe alternative for patients experiencing prolonged pain crisis in refractory TN.

RNM has yet to be fully explored and defined that includes understanding which areas should be treated and the doses are truly ablative and needed to achieve RNM. Although suspected ablative doses were used in most patients, this series provides a clinical proof of concept of the consistency of RNM in obtaining quick pain relief. Further research is needed to determine doses that can provide similar results outside of what is expected to be the ablative spectrum.
